# Magnetic nanoprecipitates and interfacial spin disorder in zero-field-annealed Ni_50_Mn_45_In_5_ Heusler alloys as seen by magnetic small-angle neutron scattering^1^

**DOI:** 10.1107/S1600576722006355

**Published:** 2022-07-15

**Authors:** Mathias Bersweiler, Philipp Bender, Inma Peral, Evelyn Pratami Sinaga, Dirk Honecker, Diego Alba Venero, Ivan Titov, Andreas Michels

**Affiliations:** a Department of Physics and Materials Science, Université du Luxembourg, 162A avenue de la Faïencerie, Luxembourg L-1511, Grand Duchy of Luxembourg; b Heinz Maier-Leibnitz Zentrum, Technische Universität München, Garching D-85748, Germany; c ISIS Neutron and Muon Facility, Science and Technology Facilities Council, Rutherford Appleton Laboratory, Chilton OX11 0QX, United Kingdom; University of Cologne, Germany

**Keywords:** magnetic neutron scattering, small-angle neutron scattering, magnetic structures, materials science, Heusler alloys

## Abstract

Shell ferromagnetism is a new functional property of certain off-stoichiometric Ni–Mn–In Heusler alloys, with a potential application in non-volatile magnetic memories and recording media. One key challenge in this field remains the determination of the structural and magnetic properties of the nanoprecipitates that are the result of an annealing-induced segregation process. Thanks to its unique mesoscopic length scale sensitivity, magnetic small-angle neutron scattering appears to be a powerful technique to disclose the microstructure of such annealing-induced nanoprecipitates. In this study, the microstructure of a zero-field-annealed off-stoichiometric Ni_50_Mn_45_In_5_ Heusler alloy is investigated by unpolarized magnetic small-angle neutron scattering. The neutron data analysis reveals a significant spin-misalignment scattering, which is mainly related to the formation of annealing-induced ferromagnetic nanoprecipitates in an antiferromagnetic matrix. These particles represent a source of perturbation which, due to dipolar stray fields, gives rise to canted spin moments in the surroundings of the particle–matrix interface. The presence of anticorrelations in the computed magnetic correlation function reflects the spatial perturbation of the magnetization vector around the nanoprecipitates. The magnetic field dependence of the zero crossing and the minima of the magnetic correlation function are qualitatively explained using the law of approach to ferromagnetic saturation for inhomogeneous spin states. More specifically, at remanence, the nanoprecipitates act magnetically as one superdefect with a correlation length that lies outside the experimental *q* range, whereas near saturation the magnetization distribution follows each individual nanoprecipitate. Analysis of the neutron data yields an estimated size of 30 nm for the spin-canted region and a value of about 75 nm for the magnetic core of the individual nanoprecipitates.

## Abstract 2

Magnetic-field-dependent small-angle neutron scattering is employed to disclose the zero-field annealing-induced spin disorder around magnetic nanoprecipitates in an off-stoichiometric Ni_50_Mn_45_In_5_ Heusler alloy.

## 1. Introduction

Over the past few decades off-stoichiometric bulk Ni_50_Mn_50−*x*_In*_x_* Heusler alloys have raised a lot of interest because of their multifunctional properties. Among the most well known and best studied properties are the magneto­caloric effect (Liu *et al.*, 2012 ▸), magnetic shape-memory effects (Umetsu *et al.*, 2009 ▸), the giant Hall effect (Dubenko *et al.*, 2009 ▸), large magnetoresistance (Yu *et al.*, 2006 ▸; Sharma *et al.*, 2006 ▸) and exchange bias effects (Pathak *et al.*, 2009 ▸; Wang *et al.*, 2011 ▸). These phenomena originate from the structural and magnetic phase transition that lies in a narrow composition range between a high-temperature cubic austenite phase with long-range ferromagnetic (FM) correlations and a low-temperature modulated/nonmodulated tetragonal martensite phase with short-range frustrated antiferromagnetic (AF) correlations (Krenke *et al.*, 2006 ▸; Ito *et al.*, 2007 ▸).

More recently, a new functionality, termed shell ferromagnetism, was discovered in the martensitic Heusler Ni_50_Mn_45_In_5_ alloy (Çakır *et al.*, 2016 ▸), and later on extended to all off-stoichiometric Ni_50_Mn_50−*x*_In*_x_* alloys with *x* up to 25 at.% (Çakır *et al.*, 2017 ▸). When annealed at high temperatures (650–750 K) and under a magnetic field (of up to 9 T), the compound segregates into predominantly cubic FM nanosized (Heusler) Ni_50_Mn_25_In_25_ precipitates that are embedded in a tetragonal AF Ni_50_Mn_50_ matrix. During the annealing treatment, the spins at the interface with the AF matrix align parallel to the field direction. Then, on removal of the magnetic field, due to the magnetic proximity effect, the interfacial spins remain strongly ferromagnetically pinned along their original direction (*i.e.* along the magnetic field direction applied during the annealing), forming the shell ferromagnet structure. Furthermore, X-ray diffraction analysis revealed that the size of the precipitates is strongly influenced by the annealing temperature and the annealing time (Dincklage *et al.*, 2018 ▸).

Note that in all of the above-described work the evidence for shell ferromagnetism was obtained rather indirectly on the basis of magnetization and X-ray diffraction measurements. A key challenge in this field remains therefore the determination of the structural and magnetic properties of the nanoprecipitates generated by the segregation process using a more direct approach (*e.g.* via Lorentz transmission electron microscopy or small-angle X-ray or neutron scattering). Very recently, polarized small-angle neutron scattering (SANS) analysis of field-annealed off-stoichiometric Ni_50_Mn_45_In_5_ Heusler alloys has suggested the presence of FM nanoprecipitates of ∼55 nm size surrounded by a magnetically inhomogeneous region with a size of about 20 nm (Benacchio *et al.*, 2019 ▸).

The purpose of the present work is to clarify the magnetic microstructure of a zero-field-annealed off-stoichiometric Ni_50_Mn_45_In_5_ Heusler compound. In particular, we study the magnetic field and temperature dependence of the magnetic properties using DC magnetization measurements combined with magnetic-field-dependent unpolarized SANS. Magnetic SANS appears to be well suited to obtaining bulk-averaged information on the structural and magnetic microstructure of off-stoichiometric martensitic Heusler alloys since it provides information about the perturbation of the magnetization vector field on a mesoscopic length scale of about 1–300 nm. For instance, for these types of system, magnetic SANS has been successfully employed to disclose the interplay between the nuclear and magnetic microstructure (Runov *et al.*, 2001 ▸, 2003 ▸, 2004 ▸, 2006 ▸) and to reveal the presence of nanometre-sized spin clusters in martensitic Ni–Mn-based Heusler alloys (Bhatti *et al.*, 2012 ▸; El-Khatib *et al.*, 2019 ▸; Sarkar *et al.*, 2020 ▸). For a summary of the fundamentals and the most recent applications of the magnetic SANS technique, we refer the reader to the review by Mühlbauer *et al.* (2019 ▸) and to the book by Michels (2021 ▸).

The paper is organized as follows. Section 2 provides some details about the sample synthesis process, the sample characterization and the neutron experiment. Section 3 gives a brief overview of the main expression for the unpolarized magnetic SANS cross section and describes the different neutron data analysis procedures to determine the purely magnetic SANS cross section and the underlying magnetic correlation function. Section 4 presents and discusses the experimental results, while Section 5 summarizes the main findings of this study. Appendix *A* [App appa] displays the experimental 2D total (nuclear + magnetic) SANS cross sections from which the 1D SANS cross sections have been determined. Appendix *B* [App appb] provides additional information on the procedure employed to estimate the nuclear scattering contribution. Finally, Appendix *C* [App appc] summarizes the 1D total (nuclear + magnetic) SANS cross sections, the purely magnetic SANS cross sections, and the computed magnetic distance distribution functions.

## 2. Experimental

The synthesis procedure for the preparation of the off-stoichiometric Ni_50_Mn_45_In_5_ polycrystalline alloy (nominal composition) used in this study is similar to that described by Benacchio *et al.* (2019 ▸). It consists of a three-step process: (i) the sample was prepared by arc-melting from high-purity elements (99.9%), followed by (ii) annealing under an Ar atmosphere at 1073 K in a sealed quartz tube for 5 days (and then quenching in water at room temperature), and finally (iii) the specimen was post-annealed in zero applied magnetic field at 700 K for 12 h, which is expected to result in an equilibrium microstructure. The thickness of the sample was estimated to be ∼0.585 mm and the sample’s surface area was determined as ∼0.248 cm^2^.

**Figure 1:**
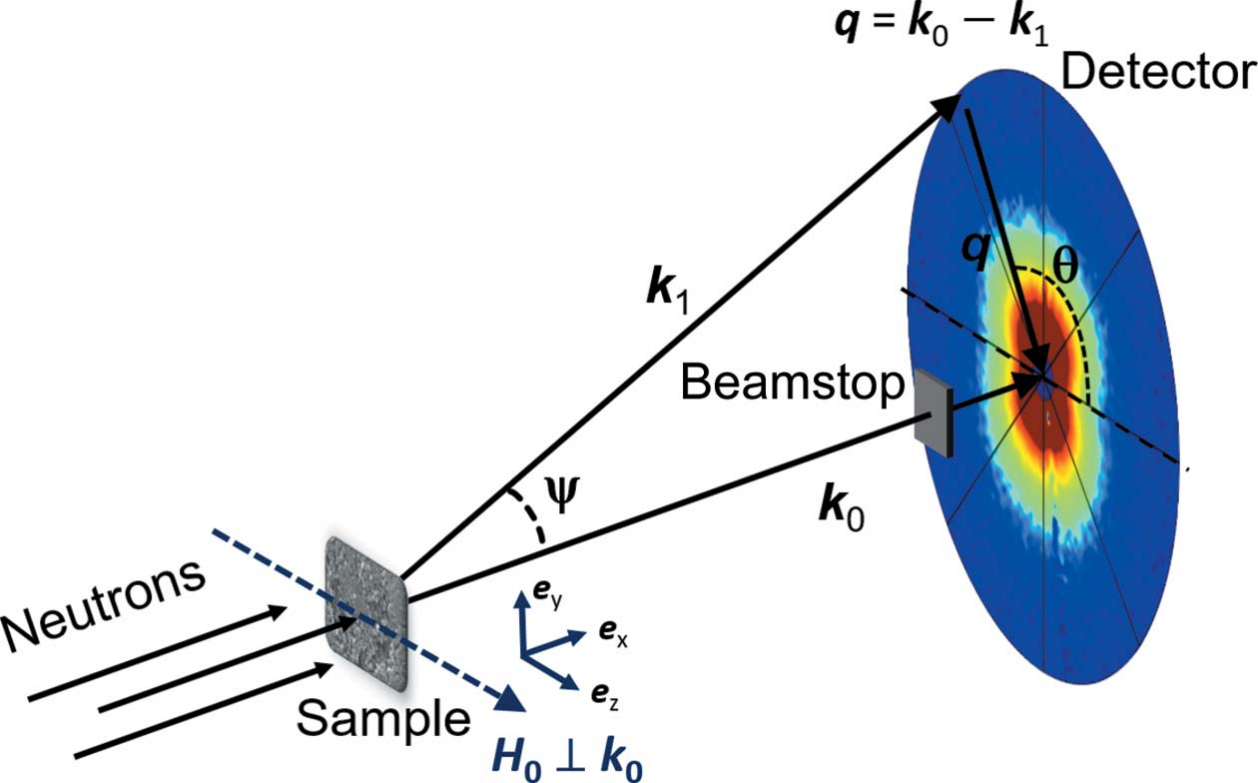
A sketch of the magnetic SANS setup. The momentum transfer vector **q** corresponds to the difference between the wavevectors of the incident (**k**_0_) and scattered (**k**_1_) neutrons, *i.e.* **q** = **k**_0_ − **k**_1_. The magnetic field **H**_0_ is applied perpendicular to the incident neutron beam, *i.e.* 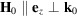 . For small-angle scattering, *i.e.* 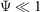 , the component *q*_*x*_ of **q** is smaller than the other two components *q*_*y*_ and *q*_*z*_, so that only correlations in the plane perpendicular to the incident neutron beam are probed.

The structural properties were determined by wide-angle X-ray diffraction (XRD) using a Bruker D8 DISCOVER dif­fractometer in Bragg–Brentano geometry (Cu *K*α radiation). Magnetization measurements were performed using a Cryogenic Ltd vibrating sample magnetometer equipped with a 14 T superconducting magnet. The neutron measurements were conducted at the ZOOM instrument at the ISIS neutron and muon source (United Kingdom). Fig. 1 ▸ depicts the experimental SANS setup used for this study. The measurements were done in time-of-flight mode using a white incident neutron beam. The neutron experiments were conducted at selected temperatures of 280, 250, 200, 150, 50 and 10 K and within a *q* range of about 0.04 ≤ *q* ≤ 0.90 nm^−1^ [*q* = (4π/λ)sin(Ψ/2), where Ψ is the scattering angle and λ is the wavelength of the incident radiation]. A magnetic field **H**_0_ was applied perpendicular to the incident neutron beam 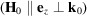 . The neutron data at each selected temperature were recorded by reducing the magnetic field from 7 T (maximum field available) down to 0 T following the magnetization curves (see Fig. 3). The neutron data reduction (corrections for background scattering and transmission) was carried out using the standard procedure implemented in the *Mantid* software (Arnold *et al.*, 2014 ▸).

## 3. Neutron data analysis

### 3.1. Unpolarized SANS cross section

For the scattering geometry where the magnetic field **H**_0_ is applied perpendicular to the incident neutron beam (see Fig. 1 ▸), the elastic unpolarized total (nuclear + magnetic) SANS cross section dΣ/dΩ at momentum-transfer vector **q** can be written as (Michels, 2021 ▸)

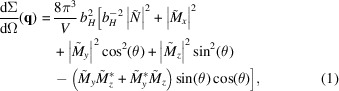

where *V* is the scattering volume, *b*_*H*_ = 2.91 × 10^8^ A^−1^ m^−1^ relates the atomic magnetic moment to the atomic magnetic scattering length, 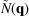 and 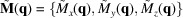 represent the Fourier transforms of the nuclear scattering length density *N*(*r*) and of the magnetization vector field **M**(**r**) = {*M_x_* (**r**), *M_y_* (**r**), *M_z_* (**r**)}, respectively, θ specifies the angle between **H**_0_ and **q** ≃ *q*{0, sin(θ), cos(θ)} in the small-angle approximation, and the asterisks * denote the complex conjugated quantities. Equation (1) shows that for a fully saturated material (*M_x_* = *M_y_* = 0) the SANS signal (in the perpendicular scattering geometry) exhibits the well known sin^2^(θ) anisotropy, provided that there exists a contrast in the longitudinal magnetization component *M_z_* (**r**) on the length scale probed by SANS. Away from saturation, more complicated angular anisotropies may be expected due to the contribution of the transverse magnetization Fourier components.

In our neutron data analysis below, we subtracted the azimuthally averaged (over ±10°) 1D SANS cross section along the field direction (**q** || **H**_0_) and at the largest available field of 7 T (approach-to-saturation regime; compare Fig. 3) from the azimuthally averaged (over 2π radians) 1D SANS cross section measured at lower fields.^2^ This subtraction procedure eliminates the (field-independent) nuclear SANS contribution 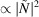 and yields the following 1D purely magnetic SANS cross section dΣ_mag_/dΩ,

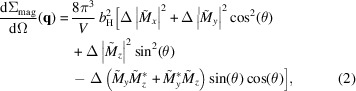

where the Δ stand for the differences between the Fourier components at the two fields considered (low-field data minus the 7 T data). We emphasize that dΣ_mag_/dΩ is strongly dominated by the two transverse magnetization Fourier components 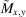 .

### 3.2. Magnetic correlation function

Generally, for a ferromagnetic material the magnetic correlation function reflects the spatial variation (perturbation) of the perpendicular magnetization components around microstructural defects (such as the precipitates studied here). Increasing the applied field results in the suppression of the amplitude and of the range of the spin-misalignment correlations around the defect. In this study, the real-space 1D magnetic distance distribution function *P*_mag_(*r*) = *r* ^2^ *C*_mag_(*r*), with *C*_mag_(*r*) being the magnetic correlation function, has been computed numerically from the experimental 1D purely magnetic SANS cross section dΣ_mag_/dΩ via an indirect Fourier transform (IFT) (Glatter, 1977 ▸). In this case, the *N*-dimensional vector *P*_mag_(*r*) is determined by minimizing the function

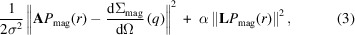

where σ = σ(*q*) is the standard deviation of each data point, **A** is the data transfer matrix, which in the case of 1D IFT has the elements *A*_*ij*_ = 4π[sin(*q*_*i*_ *r*_*j*_)/*q*_*i*_ *r*_*j*_]Δ*r*_*j*_, and **L** is a regularization matrix that is multiplied by the regularization parameter α. The numerical inversion method has already been used successfully in several other studies, *e.g.* to investigate the structural and magnetic properties of nanoparticles (Bender, Balceris *et al.*, 2017 ▸; Bender, Bogart *et al.*, 2017 ▸) and to extract the underlying 2D magnetic correlations in a nanocrystalline bulk ferromagnet (Bender *et al.*, 2021 ▸).

The full Python script used to perform the IFT and extract the 1D correlation function from the 1D SANS data can be found on the Github repository (https://github.com/PBenderLux/Data-analysis).

## 4. Results and discussion

**Figure 2:**
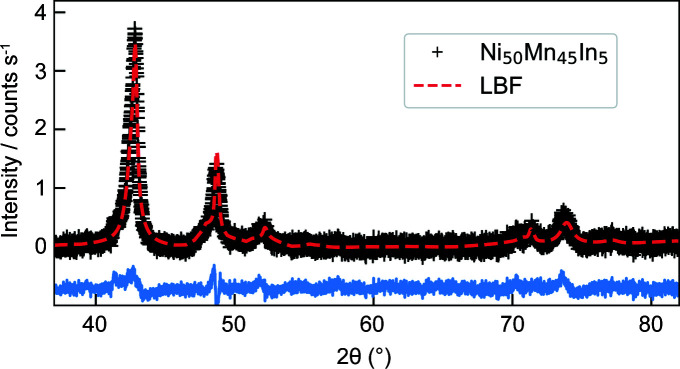
XRD pattern for Ni_50_Mn_45_In_5_ alloy (Cu *K*α radiation) (black crosses). The red dashed line shows the XRD data refinement using the Le Bail fit (LBF) method implemented in the *FULLPROF* software (Rodríguez-Carvajal, 1993 ▸) and considering two tetragonal phases in the space group *I*4/*mmm*. The bottom blue solid line represents the difference between the calculated and experimental intensities.

Fig. 2 ▸ presents the wide-angle XRD results for the Ni_50_Mn_45_In_5_ alloy. The XRD pattern features broad reflections, which can be ascribed to two crystallographic phases: the tetragonal antiferromagnetic Ni_50_Mn_50_ phase (*I*4/*mmm*, *a* ≃ 2.64 Å, *c* ≃ 3.50 Å) with an average crystallite size of about 50 nm, and the initial tetragonal Ni_50_Mn_45_In_5_ phase (*I*4/*mmm*, *a* ≃ 3.77 Å, *c* ≃ 7.0 Å) with an estimated particle size of 8 nm. As discussed in the *Introduction* [Sec sec1], these findings suggest that the phase separation at 700 K is not fully completed after 12 h of annealing and some initial phase remains intact, in agreement with the results of Çakır *et al.* (2016 ▸) and Dincklage *et al.* (2018 ▸). However, in contrast to the previous studies, the cubic Ni_50_Mn_25_In_25_ phase (*i.e.* the Heusler nanoprecipitates) cannot be detected in the XRD pattern. As discussed by Benacchio *et al.* (2019 ▸), a possible explanation for this could be the presence of a few large grains and/or a strongly bimodal particle-size distribution in the sample, which could prevent the detection of small quantities of nanoprecipitates by XRD. Note that the lattice parameter values obtained using the Le Bail fit (LBF) method for the tetragonal Ni_50_Mn_50_ phase are in line with the ones reported in the International Centre for Diffraction Data (ICDD; http://www.icdd.com) database (ICDD card No 01-071-9643). No record could be found in the ICDD database for the initial Ni_50_Mn_45_In_5_ phase. Further complementary structural characterization methods, such as electron microscopy or neutron diffraction (as a function of annealing time and temperature), are required to obtain additional information on the microstructure of the zero-field-annealed Ni_50_Mn_45_In_5_ alloy, in particular regarding here the observed absence of the cubic Ni_50_Mn_25_In_25_ phase.

**Figure 3:**
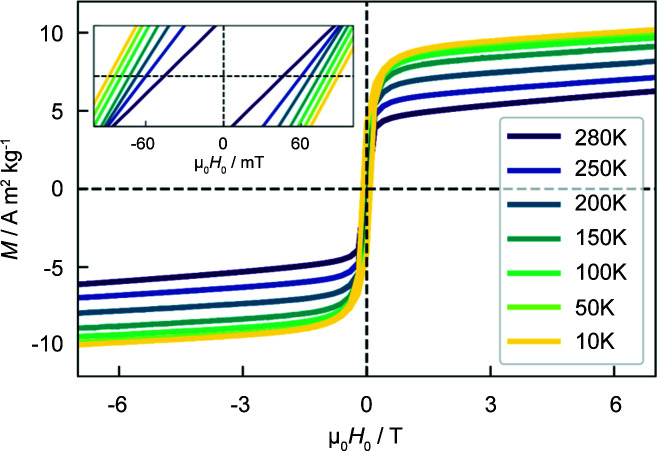
Magnetization curves for the Ni_50_Mn_45_In_5_ alloy measured at the selected temperatures of 280, 250, 200, 150, 100, 50 and 10 K. (Inset) An enlargement of the magnetization curves between ±100 mT.

Fig. 3 ▸ displays the magnetization curves of the Ni_50_Mn_45_In_5_ alloy at different temperatures. The magnetization does not saturate even at the highest applied magnetic field values, and exhibits a hysteresis with a coercive field that increases with decreasing temperature from 46 mT at 280 K to 88 mT at 10 K (see inset in Fig. 3 ▸). The presence of both features points to the coexistence of a paramagnetic-like and ferromagnetic contribution, respectively. As previously reported (Çakır *et al.*, 2016 ▸, 2017 ▸), the emergence of a ferromagnetic-like behaviour in Ni_50_Mn_45_In_5_ strongly suggests the formation of dominantly ferromagnetic nanoprecipitates in an AF Ni–Mn matrix. From the estimated saturation magnetization at 10 K [*M*_S_ = 11.1 A m^2^ kg^−1^, determined from a linear fit of *M*(1/*H*_0_) for high field values] and the specific magnetization analysis detailed by Çakır *et al.* (2016 ▸), we estimate that about 60% of the Ni_50_Mn_45_In_5_ sample has been decomposed into Ni_50_Mn_25_In_25_. This result therefore supports the hypothesis of an incomplete phase separation process, as already suggested by the XRD results. Moreover, the absence of a vertical shift in the magnetization curves (found in the field-annealed samples) indicates the (expected) absence of interfacial spin ordering between the AF matrix and the FM nanoprecipitates during nucleation and growth, as has been already discussed by Çakır *et al.* (2016 ▸, 2017 ▸).

**Figure 4:**
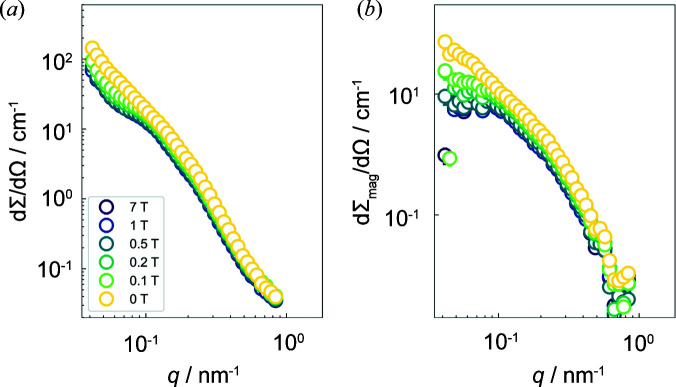
(*a*) The magnetic field dependence of the azimuthally averaged (over 2π radians) total (nuclear + magnetic) SANS cross section dΣ/dΩ at the selected temperature of 280 K. (*b*) The corresponding purely magnetic SANS cross section dΣ_mag_/dΩ (log–log scales).

Fig. 4 ▸(*a*) shows the magnetic field dependence of the azimuthally averaged (over 2π radians) total (nuclear + magnetic) SANS cross section dΣ/dΩ at the selected temperature of 280 K. The cross section dΣ/dΩ exhibits a broad shoulder at intermediate momentum transfers *q* and a weakly magnetic field dependent intensity at the smallest *q*. Since the nuclear scattering is field independent, the magnetic field dependence of dΣ/dΩ can only result from the magnetic scattering. Fig. 4 ▸(*b*) presents the corresponding purely magnetic SANS cross section dΣ_mag_/dΩ, which was obtained using the specific neutron data procedure developed in Section 3.1. The strong field dependence of dΣ_mag_/dΩ at small *q* is due to spin-misalignment scattering, *i.e.* it results from the failure of the spins to be completely aligned along **H**_0_. In this way (via the subtraction procedure), the broad shoulder at intermediate *q* and the magnetic field dependence of the SANS signal at the smallest *q* become more clearly visible. These particular magnetic features, in addition to the sin^2^(θ)-type angular anisotropy due to longitudinal magnetization fluctuations [compare equation (1)] observed in the 2D SANS patterns (see Appendix *A* [App appa]), were also observed in field-annealed Ni_50_Mn_45_In_5_ samples. The origin of these three features in the SANS signal has been attributed to the formation of annealing-induced ferromagnetic nanoprecipitates in the AF matrix (Benacchio *et al.*, 2019 ▸). More specifically, the particles represent a source of perturbation, which gives rise to canted spin moments in the surroundings of the particle–matrix interface, *e.g.* via inhomogeneous dipolar stray fields and/or strain fields that are related to the jump in the magnetization in the vicinity of the particle–matrix interface.

**Figure 5:**
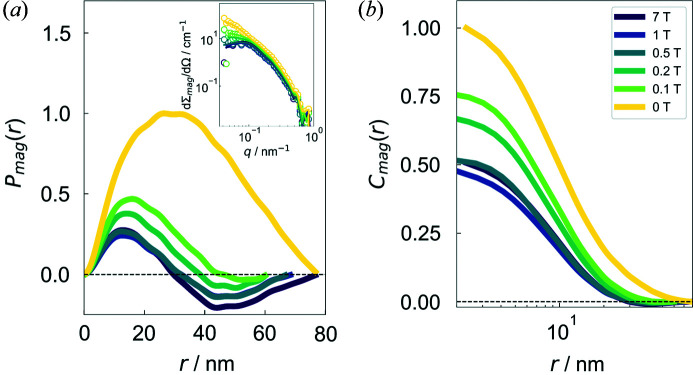
(*a*) The field dependence of the magnetic distance distribution function *P*_mag_(*r*) at 280 K. The *P*_mag_(*r*) values were computed numerically via an indirect Fourier transform (IFT) of the experimental dΣ_mag_/dΩ data shown in Fig. 4 ▸(*b*). Note that for comparison with the results obtained at lower temperatures, as summarized in Appendix *C* [App appc], the *P*_mag_ at each field has been normalized by the maximum peak intensity value obtained at remanence. (Inset) A comparison between the experimental dΣ_mag_/dΩ plotted in Fig. 4 ▸(*b*) (coloured open circles) and the reconstructed dΣ_mag_/dΩ based on the IFT of the numerically computed *P*_mag_ shown in Fig. 5(*a*) (coloured solid lines). (*b*) The corresponding magnetic correlation function *C*_mag_(*r*) = *P*_mag_(*r*)/*r* ^2^ (semi-logarithmic sale).

**Figure 6:**
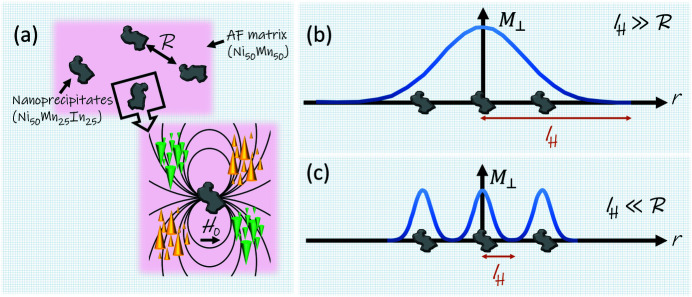
(*a*) A simplified sketch of the spin-misalignment correlations around defects in an antiferromagnetic matrix. The jump in the magnetization magnitude at the interface between the precipitate and the matrix gives rise to a dipolar stray field (drawn here with spherical symmetry for simplicity), which itself exerts a torque on the matrix spins and in this way produces spin disorder and a concomitant field-dependent contrast for magnetic SANS. *R* denotes the average distance between defects. Coloured arrows represent the magnetization component 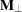 perpendicular to **H**_0_. (*b*), (*c*) Illustrations of the magnetization distribution around defects for the cases of *l*_*H*_ ≫ *R* (low field) and 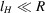 (large field), respectively. Adapted from Kronmüller & Seeger (1961 ▸).

Fig. 5 ▸(*a*) presents the magnetic field dependence of the numerically computed magnetic distance distribution function *P*_mag_ at 280 K. The *P*_mag_ profiles are strongly affected by the applied magnetic field strength. In particular, by reducing the magnetic field, we see that (i) the oscillatory behaviour with negative *P*_mag_ values vanishes, and (ii) the zero crossings shift to larger *r* values. As previously discussed (Benacchio *et al.*, 2019 ▸), here the origin of the negative part of the oscillatory *P*_mag_ may be attributed to the spatial nanometre-scale variation in the orientation of the magnetic moments around the annealing-induced ferromagnetic nanoprecipitates, rather than to an inhomogeneous core–shell-type structure (Lang & Glatter, 1996 ▸). More precisely, for a dipolar-type perturbation such as the one sketched in Fig. 6 ▸(*a*), the transverse magnetization components (*M_x_*, *M_y_*) change their sign in the direction of the applied field, in this way giving rise to negative values of the distance distribution function (termed anti­correlations). The zero crossings and the minima of *P*_mag_ are dependent on the applied field **H**_0_. Fig. 5 ▸(*b*) depicts the corresponding correlation function *C*_mag_ = *P*_mag_/*r* ^2^. Due to the *r* ^2^ factor, features in *P*_mag_ at medium and large distances are more pronounced than in *C*_mag_. In agreement with the functional behaviour of *P*_mag_(*r*), we see that increasing the field results in a reduction in the amplitude and range of the spin-misalignment correlations. The *C*_mag_(*r*) shapes agree qualitatively with the ones computed numerically by Erokhin *et al.* (2015 ▸) for a nanoporous dipolar stray-field-dominated system, supporting therefore the existence of anticorrelations in Ni_50_Mn_45_In_5_.

The disappearance of the oscillatory character in *P*_mag_ for *r* > 30 nm with decreasing field can be qualitatively interpreted using the law of approach to ferromagnetic saturation for inhomogeneous spin states. Magnetization nonuniformities are induced by the stress fields of microstructural defects such as point defects, dislocations, grain boundaries or precipitates (Kronmüller & Fähnle, 2003 ▸). The field-dependent micromagnetic exchange length *l*_*H*_ ∝ *H* ^−1/2^ characterizes the size of these nonuniformities and can be seen as the resolution limit of the magnetization. At applied fields of the order of a few tesla, *l*_*H*_ takes on values of the order of a nanometre, and may increase to about 100 nm (and even larger) in small fields. At remanence, *l*_*H*_ becomes larger than the average distance *R* between the magnetic nanoprecipitates, so that the nano­precipitates act magnetically as one superdefect with a corresponding (large) magnetic correlation length that, in our experiment, lies outside of the available experimental *q* range. By contrast, near saturation, *l*_*H*_ becomes smaller than *R*, and the magnetization distribution then follows each individual defect (nanoprecipitate). Since their sizes are of the order of a few tens of nanometres, the correlation features in *P*_mag_ become visible within the available experimental *q* range and for the highest field (near to saturation). Fig. 6 ▸ graphically illustrates the magnetization distribution around the nanoprecipitates (defects) at low field and at a field close to saturation. This sketch should help the reader to obtain a better understanding of the physical picture behind our discussion.

Therefore, similar to our previous work (Benacchio *et al.*, 2019 ▸), from the *P*_mag_ data computed in the highest fields, one can relate the first and second zero crossings to the size δ of the magnetically inhomogeneous region surrounding the nanoprecipitates and to the size *D* of the individual nanoprecipitates. Near saturation (μ_0_ *H*_0_ = 7 T), we estimate *D* ≃ 75 nm and δ ≃ 30 nm. Note that both quantities δ and *D* seem to be temperature independent at 7 T (compare the *P*_mag_ plots in Appendix *C* [App appc]). The value for δ is slightly larger than the one determined for the 5 T field-annealed sample (Benacchio *et al.*, 2019 ▸). This increase can be easily understood by considering the role played by the magnetic field on the spin configuration at the interface between the AF matrix and the FM nanoprecipitates during the annealing process. When the annealing is realized under a magnetic field, the spins at the interface can be pinned along the field direction due to the magnetic proximity effect, whereas they remain randomly oriented in the absence of a magnetic field. Therefore, an increase in the size of the magnetically inhomogeneous region seen by magnetic SANS is expected in the case of zero-field-annealed off-stoichiometric Ni_50_Mn_45_In_5_ Heusler alloys. Furthermore, the shift of the zero crossing to smaller *r* values with increasing field strength suggests that the canted spins at the interface tend to align with respect to the magnetic field **H**_0_ (as expected).

## 5. Conclusions

We have employed X-ray diffraction, magnetometry and field-dependent unpolarized magnetic SANS to disclose the magnetic microstructure of a zero-field-annealed off-stoichiometric Ni_50_Mn_45_In_5_ Heusler alloy. X-ray diffraction reveals the presence of a nanoscale microstructure and indicates that the segregation process at 700 K is not fully completed after 12 h of annealing. The magnetometry data exhibit a ferromagnetic-like behaviour, which strongly suggests the formation of annealing-induced ferromagnetic nanoprecipitates in the antiferromagnetic matrix, as postulated by Çakır *et al.* (2016 ▸, 2017 ▸). The analysis of the field-dependent magnetic SANS data reveals a strong spin mis­alignment on the mesoscopic length scale caused by the annealing-induced ferromagnetic nanoprecipitates. In fact, the field-dependent analysis of the magnetic distance distribution function, obtained by an indirect Fourier transform technique, confirms that the nanoprecipitates are surrounded by a magnetically inhomogeneous region where the canted spins tend to align with respect to the magnetic field. Near magnetic saturation, the size of the spin-canted region and the size of the individual nanoprecipitates have been estimated to ∼30 and ∼75 nm, respectively. The presented neutron data analysis (in Fourier and real space) is particularly useful for studying the nanoscale magnetic inhomogeneities of bulk materials at the mesoscopic length scale. Finally, it demonstrates that unpolarized magnetic SANS might be a practicable alternative to time-consuming and low-intensity polarized neutron measurements.

## Figures and Tables

**Figure 7:**
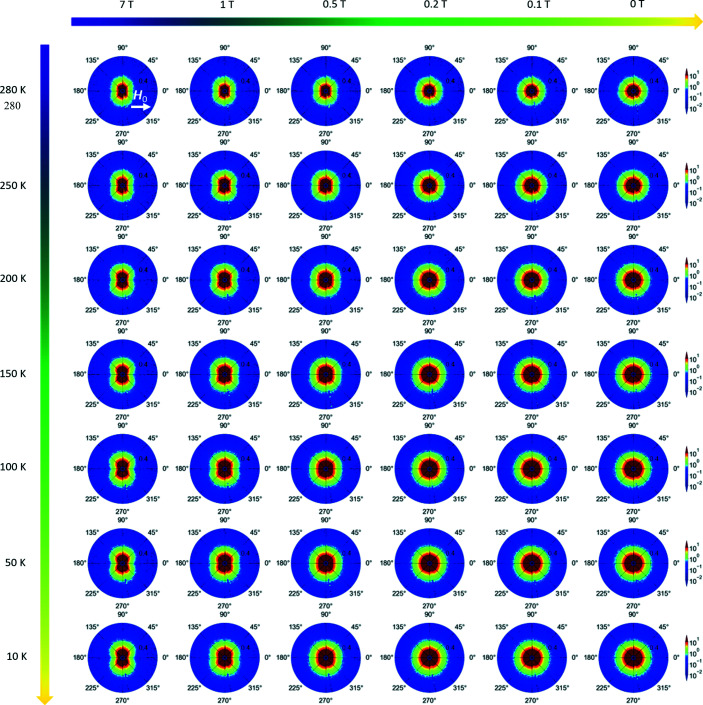
The magnetic field and temperature dependence of the experimental 2D total (nuclear + magnetic) SANS cross section dΣ/dΩ. The applied magnetic field is horizontal in the plane of the detector 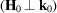 . Note that the dΣ/dΩ are plotted in polar coordinates with *q* in nm^−1^, θ in degrees and the intensity in cm^−1^.

**Figure 8:**
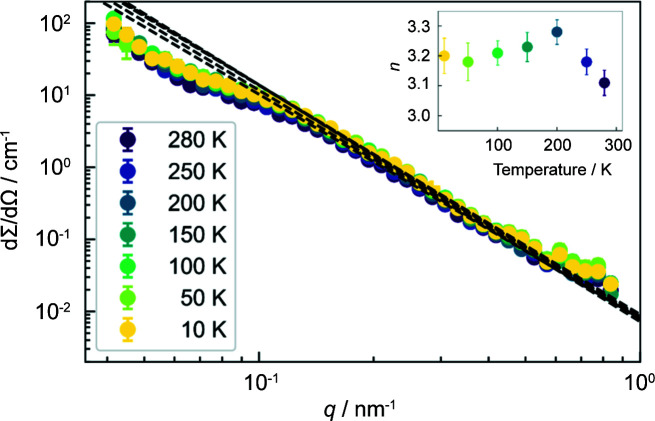
The temperature dependence of the azimuthally averaged (over ±10°) 1D total (nuclear + magnetic) SANS cross section dΣ/dΩ measured along the field direction (**q** || **H**_0_) and at the highest applied field value of 7 T (log–log scale). Dashed lines show the extrapolation of dΣ/dΩ ∝ *q* ^−*n*^. The Porod fits were restricted to 0.25 ≤ *q* ≤ 0.46 nm^−1^. (Inset) The temperature dependence of the asymptotic power-law exponent *n*.

**Figure 9:**
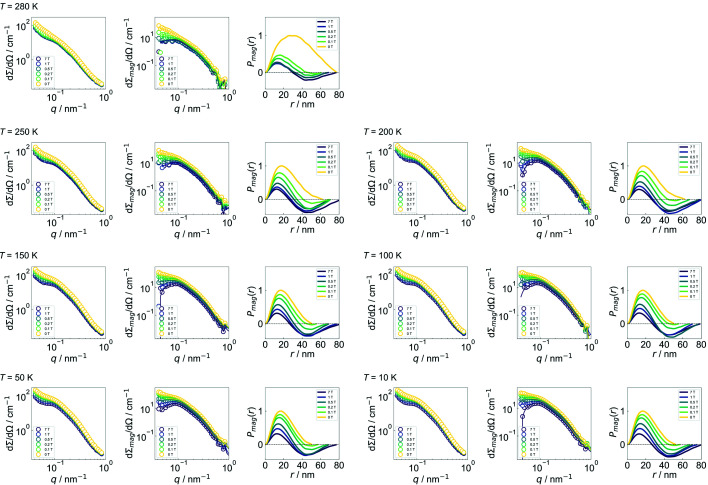
The magnetic field and temperature dependence of the experimental azimuthally averaged (over 2π radians) 1D total (nuclear + magnetic) SANS cross sections dΣ/dΩ, the corresponding purely magnetic SANS cross sections dΣ_mag_/dΩ and the magnetic distance distribution functions *P*_mag_. The *P*_mag_ values were computed numerically via an indirect Fourier transform of the experimental dΣ_mag_/dΩ data based on equation (3). The *P*_mag_ value at each field has been normalized by the maximum peak intensity value obtained at remanence.

## Footnotes

2 The 2D total (nuclear + magnetic) SANS cross sections from which the azimuthally averaged (over 2π radians and over ±10°) 1D SANS cross sections have been determined are summarized in Appendix *A* [App appa]. Additional information about the determination of the nuclear SANS cross section is provided in Appendix *B* [App appb].

## Appendix A Two-dimensional total SANS cross sections

In this appendix, we provide the experimental 2D total (nuclear + magnetic) SANS cross sections dΣ/dΩ, from which the azimuthally averaged (over 2π radians and over ±10°) 1D SANS cross sections have been determined.

As can be seen in Fig. 7 ▸, in the temperature range of 10–280 K and at the highest applied field of 7 T, the 2D neutron patterns are predominantly elongated perpendicular to the magnetic field direction. This particular feature is the signature of the sin^2^(θ)-type angular anisotropy [compare equation (1)]. At remanence, the patterns are isotropic. Since for an ideal defect-free AF the magnetization within a mesoscopic volume is zero, the magnetic scattering contrast would vanish. Therefore, the magnetic field dependence of the 2D SANS patterns strongly suggests the presence of an effectively ferromagnetic component in dΣ/dΩ, which is compatible with the formation of annealing-induced ferromagnetic precipitates in the AF matrix.

## Appendix B Estimation of nuclear SANS cross section

The purpose of this appendix is to provide additional information about the procedure used here to estimate the nuclear SANS cross section dΣ_nuc_/dΩ. As detailed in Section 3.1, to separate the purely magnetic SANS cross section dΣ_mag_/dΩ from the total (nuclear + magnetic) dΣ/dΩ, it is necessary to determine dΣ_nuc_/dΩ.

Fig. 8 ▸ presents the temperature dependence of the azimuthally averaged (over ±10°) 1D total (nuclear + magnetic) SANS cross section dΣ/dΩ measured along the field direction (**q** || **H**_0_) at the highest applied magnetic field of 7 T. The total dΣ/dΩ are only weakly temperature dependent within the experimental *q* range. Noting moreover that the sample is in the approach-to-saturation regime at 7 T (compare Fig. 3 ▸), we believe that the dΣ/dΩ data shown in Fig. 8 ▸ are a good approximation for the nuclear SANS cross section dΣ_nuc_/dΩ. We also remind the reader that the paramagnetic-like magnetization contribution related to the antiferromagnetic matrix is always present within the shown field range. The asymptotic power-law exponent *n* in dΣ/dΩ ∝ *q* ^−*n*^ was found to be weakly temperature dependent and smaller than the Porod value of *n* = 4 (see the inset of Fig. 8 ▸). Similar observations have also been reported in Heusler-derived multiferroic alloys (Bhatti *et al.*, 2012 ▸). As discussed by those authors, the nature of the Porod scattering changes in such alloys and might be related to the magnetic ground state in the martensitic phase.

## Appendix C One-dimensional SANS cross sections and magnetic distance distribution functions

Fig. 9 ▸ summarizes the experimental azimuthally averaged (over 2π radians) 1D total (nuclear + magnetic) SANS cross sections dΣ/dΩ, the corresponding purely magnetic SANS cross sections dΣ_mag_/dΩ and the magnetic distance distribution functions *P*_mag_ using the neutron data analysis detailed in Section 3.

## References

[bb1] Arnold, O., Bilheux, J. C., Borreguero, J. M., Buts, A., Campbell, S. I., Chapon, L., Doucet, M., Draper, N., Ferraz Leal, R., Gigg, M. A., Lynch, V. E., Markvardsen, A., Mikkelson, D. J., Mikkelson, R. L., Miller, R., Palmen, K., Parker, P., Passos, G., Perring, T. G., Peterson, P. F., Ren, S., Reuter, M. A., Savici, A. T., Taylor, J. W., Taylor, R. J., Tolchenov, R., Zhou, W. & Zikovsky, J. (2014). *Nucl. Instrum. Methods Phys. Res. A*, **764**, 156–166.

[bb2] Benacchio, G., Titov, I., Malyeyev, A., Peral, I., Bersweiler, M., Bender, P., Mettus, D., Honecker, D., Gilbert, E. P., Coduri, M., Heinemann, A., Mühlbauer, S., Çakır, A., Acet, M. & Michels, A. (2019). *Phys. Rev. B*, **99**, 184422.

[bb3] Bender, P., Balceris, C., Ludwig, F., Posth, O., Bogart, L. K., Szczerba, W., Castro, A., Nilsson, L., Costo, R., Gavilán, H., González-Alonso, D., Pedro, I., Barquín, L. F. & Johansson, C. (2017). *New J. Phys.* **19**, 073012.

[bb4] Bender, P., Bogart, L. K., Posth, O., Szczerba, W., Rogers, S. E., Castro, A., Nilsson, L., Zeng, L. J., Sugunan, A., Sommertune, J., Fornara, A., González-Alonzo, D., Barquín, L. F. & Johansson, C. (2017). *Sci. Rep.* **7**, 46990. 10.1038/srep45990 PMC5387715 28397851

[bb5] Bender, P., Leliaert, J., Bersweiler, M., Honecker, D. & Michels, A. (2021). *Small Sci.* **1**, 2000003. 10.1002/smsc.202000003 PMC11935939 40212415

[bb6] Bhatti, K. P., El-Khatib, S., Srivastava, V., James, R. D. & Leighton, C. (2012). *Phys. Rev. B*, **85**, 134450.

[bb8] Çakır, A., Acet, M. & Farle, M. (2016). *Sci. Rep.* **6**, 28931. 10.1038/srep28931 PMC4944126 27412644

[bb9] Çakır, A., Acet, M., Wiedwald, U., Krenke, T. & Farle, M. (2017). *Acta Mater.* **127**, 117–123.

[bb10] Dincklage, L., Scheibel, F., Çaklr, A., Farle, M. & Acet, M. (2018). *AIP Adv.* **8**, 025012.

[bb11] Dubenko, I., Pathak, A. K., Stadler, S., Ali, N., Kovarskii, Y., Prudnikov, V. N., Perov, N. S. & Granovsky, A. B. (2009). *Phys. Rev. B*, **80**, 092408.

[bb12] El-Khatib, S., Bhatti, K. P., Srivastava, V., James, R. D. & Leighton, C. (2019). *Phys. Rev. Mater.* **3**, 104413.

[bb13] Erokhin, S., Berkov, D. & Michels, A. (2015). *Phys. Rev. B*, **92**, 014427. 10.1103/PhysRevLett.114.149701 25910167

[bb14] Glatter, O. (1977). *J. Appl. Cryst.* **10**, 415–421.

[bb15] Ito, W., Imano, Y., Kainuma, R., Sutou, Y., Oikawa, K. & Ishida, K. (2007). *Metall. Mater. Trans. A*, **38**, 759–766.

[bb16] Krenke, T., Acet, M., Wassermann, E. F., Moya, X., Mañosa, L. & Planes, A. (2006). *Phys. Rev. B*, **73**, 174413.

[bb17] Kronmüller, H. & Fähnle, M. (2003). *Micromagnetism and the Microstructure of Ferromagnetic Solids.* Cambridge University Press.

[bb18] Kronmüller, H. & Seeger, A. (1961). *J. Phys. Chem. Solids*, **18**, 93–115.

[bb19] Lang, P. & Glatter, O. (1996). *Langmuir*, **12**, 1193–1198.

[bb20] Liu, J., Gottschall, T., Skokov, K. P., Moore, J. D. & Gutfleisch, O. (2012). *Nat. Mater.* **11**, 620–626. 10.1038/nmat3334 22635044

[bb21] Michels, A. (2021). *Magnetic Small-Angle Neutron Scattering: A Probe for Mesoscale Magnetism Analysis.* Oxford University Press.

[bb22] Mühlbauer, S., Honecker, D., Périgo, A., Bergner, F., Disch, S., Heinemann, A., Erokhin, S., Berkov, D., Leighton, C., Eskildsen, M. R. & Michels, A. (2019). *Rev. Mod. Phys.* **91**, 015004.

[bb23] Pathak, A. K., Khan, M., Gautam, B. R., Stadler, S., Dubenko, I. & Ali, N. (2009). *J. Magn. Magn. Mater.* **321**, 963–965.

[bb24] Rodríguez-Carvajal, J. (1993). *Physica B*, **192**, 55–69.

[bb26] Runov, V. V., Chernenkov, Y. P., Runova, M. K., Gavriljuk, V. G. & Glavatska, N. I. (2001). *JETP Lett.* **74**, 590–595.

[bb27] Runov, V. V., Chernenkov, Y. P., Runova, M. K., Gavriljuk, V. G. & Glavatska, N. I. (2003). *Physica B*, **335**, 109–113.

[bb28] Runov, V. V., Chernenkov, Y. P., Runova, M. K., Gavrilyuk, V. G., Glavatska, N. I., Goukasov, A. G., Koledov, V. V., Shavrov, V. G. & Khovaiˇlo, V. V. (2006). *J. Exp. Theor. Phys.* **102**, 102–113.

[bb25] Runov, V., Runova, M., Gavriljuk, V. & Glavatska, N. (2004). *Physica B*, **350**, E87–E89.

[bb29] Sarkar, S. K., Ahlawat, S., Kaushik, S. D., Babu, P. D., Sen, D., Honecker, D. & Biswas, A. (2020). *J. Phys. Condens. Matter*, **32**, 115801. 10.1088/1361-648X/ab5876 31739303

[bb30] Sharma, V. K., Chattopadhyay, M. K., Shaeb, K. H. B., Chouhan, A. & Roy, S. B. (2006). *Appl. Phys. Lett.* **89**, 222509.

[bb31] Umetsu, R. Y., Kusakari, Y., Kanomata, T., Suga, K., Sawai, Y., Kindo, K., Oikawa, K., Kainuma, R. & Ishida, K. (2009). *J. Phys. D Appl. Phys.* **42**, 075003.

[bb32] Wang, B. M., Liu, Y., Ren, P., Xia, B., Ruan, K. B., Yi, J. B., Ding, J., Li, X. G. & Wang, L. (2011). *Phys. Rev. Lett.* **106**, 077203. 10.1103/PhysRevLett.106.077203 21405539

[bb33] Yu, S. Y., Liu, Z. H., Liu, G. D., Chen, J. L., Cao, Z. X., Wu, G. H., Zhang, B. & Zhang, X. X. (2006). *Appl. Phys. Lett.* **89**, 162503.

